# Chemogenetic fingerprinting by analysis of cellular growth dynamics

**DOI:** 10.1186/1472-6769-8-3

**Published:** 2008-08-22

**Authors:** Jonas Warringer, Dragi Anevski, Beidong Liu, Anders Blomberg

**Affiliations:** 1 Department of Cell and Molecular biology, University of Gothenburg, Gothenburg, Sweden; 2 School of Mathematical Sciences, University of Gothenburg, Gothenburg, Sweden

## Abstract

**Background:**

A fundamental goal in chemical biology is the elucidation of on- and off-target effects of drugs and biocides. To this aim chemogenetic screens that quantify drug induced changes in cellular fitness, typically taken as changes in composite growth, is commonly applied.

**Results:**

Using the model organism *Saccharomyces cerevisiae *we here report that resolving cellular growth dynamics into its individual components, growth lag, growth rate and growth efficiency, increases the predictive power of chemogenetic screens. Both in terms of drug-drug and gene-drug interactions did the individual growth variables capture distinct and only partially overlapping aspects of cell physiology. In fact, the impact on cellular growth dynamics represented functionally distinct chemical fingerprints.

**Discussion:**

Our findings suggest that the resolution and quantification of all facets of growth increases the informational and interpretational output of chemogenetic screening. Hence, by facilitating a physiologically more complete analysis of gene-drug and drug-drug interactions the here reported results may simplify the assignment of mode-of-action to orphan bioactive compounds.

## Background

Specifying on- and off-target effects of drugs and biocides constitutes a central goal in pharmacology, ecotoxicology and chemical biology. Drugs are also used as potent inhibitors generating specific perturbations in systems biology. The overall chemotoxicity of compounds is typically measured as the growth reducing impact on organisms. Mode-of-action information of a drug can be obtained by quantifying changes in fitness of genome-wide collections of knockout strains [1-5]. Knockouts that render cells sensitive to a drug identify pathways that buffer the cell against the chemical perturbation, thereby providing clues about its mechanism of toxicity. Moreover, compounds with similar biological effects have similar chemogenetic profiles [6-8]. Thus, analysis of a compendium of chemical genetic profiles facilitates the identification of bioactive compounds with similar biological effects and the tentative assignment of biological targets to novel drugs. This approach has been successfully applied using both yeast [1-5] and bacteria [9,10]. In genome-wide chemogenetic approaches the fitness of knockouts is typically measured as changes in composite growth (as colony size on agar or end-point in culture density) or, alternatively, by competitive cultivations of pooled knockouts tagged with specific DNA sequences [5,11]. Precise quantification of composite features of growth on a smaller scale may also be achieved by long term competition of individual, fluorescently labelled strains against a reference strain labelled with a complementary fluorophore [12]. Here, we apply a high precision micro-cultivation approach [13,14] to investigate the importance of providing a detailed resolution of growth dynamics when scoring various drug effects, both as gene-drug interactions and drug-drug interactions. We show that resolving growth dynamics in the model organism *Saccharomyces cerevisiae *is in many cases essential to uncover the effects of drugs and for the functional interpretation of drug action.

## Results and discussion

### Extraction of growth variables resolves composite growth

**Figure 1:**
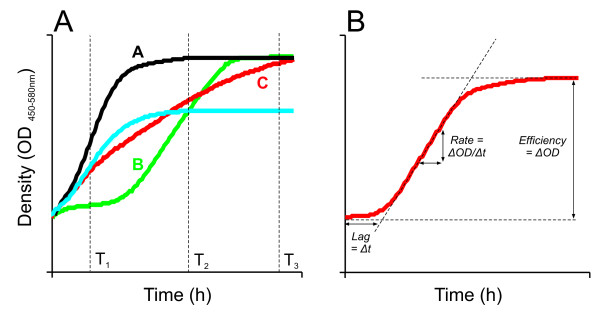
**Extraction of growth variables**. A) Extraction of the composite growth measure (density reached) at various time-points, T_1_, T_2 _and T_3_, in absence of stress (A) and in presence of a compounds that impact on growth lag (B) growth rate (C) or growth efficiency (D). B) Extraction of growth variables. Growth rate is extracted as the slope in exponential phase converted into population doubling time (h), growth lag (h) is given by the intercept of the initial density and the slope, and growth efficiency (OD units) is calculated as the total change in density for cells having reached stationary phase. Detailed descriptions of growth variable extraction may be found in earlier publications [13,14].

Living cells, tissues and populations follow a sigmoidal growth curve that is defined by the three fundamental growth variables growth lag (response time), growth rate (doubling time) and growth efficiency (gain in biomass given the available resources). However, current large scale approaches that measure drug induced changes in fitness considers a composite of these variables, as measured as cell density reached at a specified time-point, and thus do not resolve growth perturbations into its individual components. This represents a potential problem as the different growth variables may encapsulate distinct and only partially overlapping features of cell physiology (see below). Hence drugs affecting the composite growth feature similarly at a specified time (T_2_) but the individual growth variables differently, may mistakenly be suspected of having similar modes-of-action (Fig 1A). The problem is further exasperated by the dependence of the composite variable on which time point is specified, as analysis performed at different time points (T_1_, T_2_, T_3_) may lead to radically different interpretations of a drug's mode-of-action (Fig 1A). Here, we measure to what extent drugs impact on individual growth variables, whether these effects reflect drug mode-of-action and the degree of overlap between growth variables. Using a highly parallelized micro-cultivation approach we precisely quantify drug induced changes in growth dynamics and extract the three growth variables using an automated procedure [13]. Growth rate is extracted as the slope in the exponential phase converted into population doubling time (h), growth lag (h) is given by the intercept of the initial density and the slope, and growth efficiency (optical density units) is calculated as the total change in density for cultures having reached stationary phase (Fig 1B). Detailed descriptions of growth variable extraction may be found in earlier publications [13,14]. It should be observed that the extracted growth variables may be partially confounded by hard to measure features of cell death, especially at higher stress magnitudes. However, this influence should be minor given our experimental design with stress levels set to marginal growth impact.

### Impact on wild type cellular growth dynamics constitutes a distinct chemical fingerprint

**Figure 2:**
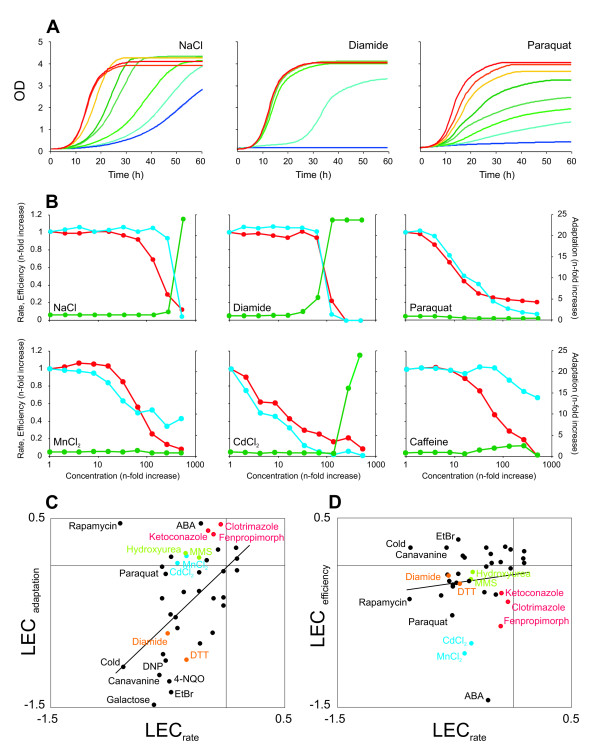
**Differential impact of bioactive compounds on cellular growth dynamics**. A) Growth of yeast WT populations in the presence of increasing doses of bioactive compounds. Concentrations of NaCl (2 M, 1.5 M, 1.2 M, 0.9 M, 0.65 M, 0.45 M, 0.3 M, 0.2 M), Diamide (1.9 mM, 1.5 mM, 1.2 mM, 1 mM, 0.8 mM, 0.6 mM, 0.45 mM, 0.3 mM), Paraquat (10 mg/mL, 5 mg/mL, 2.5 mg/mL, 1.2 mg/mL, 0.6 mg/mL, 0.3 mg/mL, 0.2 mg/mL, 0.1 mg/mL) are represented with colours, red indicating the lowest concentration, blue indicating the highest concentration. B) Dose response correlations of yeast WT populations considering growth lag (green), growth rate (red) and growth efficiency (blue), n = 2. C-D) Comparing the relative effects (LEC) of bioactive compounds on yeast WT fitness variables. Color indicates specific functional groups (red = ergosterol biosynthesis inhibitors, green = DNA damaging agents, blue = heavy metals, orange = redox status distorters). For growth lags, a cut-off at a 24-fold increase has been applied. C) Growth lag vs. growth rate. D) Growth efficiency vs. growth rate.

To investigate to what extent diverse bioactive compounds affect yeast growth dynamics we screened a set of 38 drugs that target a range of cellular processes. The chemicals encompassed both broad specificity compounds, such as NaCl and CdCl_2_, and inhibitors of distinct biological processes, such as the ribonucleotide reductase inhibitor hydroxyurea and the TOR pathway inhibitor rapamycin. Cultivating yeast wild type cells in a ladder of drug concentrations we observed a surprisingly wide variety of effects on cellular growth dynamics (Fig 2A). Dose-response correlations for the three different growth variables highlighted the functional diversity among drugs (Fig 2B). For example, the osmotic stress inducer NaCl and the cAMP phosphodiesterase inhibitor caffeine preferentially affected growth rate at low concentrations, whereas the oxidizer diamide initially affected growth lag and the heavy metals CdCl_2 _and MnCl_2 _primarily reduced the growth efficiency (Fig 2B). Although the growth rate was eventually reduced by essentially all drugs in the array, this reduction was frequently detectable only at extreme concentrations with severe impact on growth lag or growth efficiency. For example, a 20% reduction in diamide growth rate was accompanied by a 200% increase in diamide growth lag. Furthermore, the concentration dependence of the different compounds where strikingly different; while the growth lag and growth rate changed rather gradually at increasing concentrations for paraquat and CdCl_2_, distinctly steep dose-responses where recorded for the same growth variables in diamide and NaCl. Thus, dose-response curves based on high-resolution phenotyping of a wild type yeast strains constitute drug-specific chemical fingerprints.

To provide an overall view of the relative effect of the different bioactive compounds on wild type growth variables, we formed ratios (Logarithmic Environmental Coefficients, LEC) that compare growth with and without drugs. These LEC ratios were constructed at drug concentrations corresponding to a 30–75% reduction in the growth variable most affected by a drug. Care was taken to ensure that the concentration used accurately reflected the dominant drug impact (the effect observed at low drug concentrations) on cellular growth dynamics as defined by the individual dose-response profiles. Comparing LEC_rate _against LEC_adaptation _and LEC_efficiency _for the 38 compounds in the set it was clear that diverse drugs impacted differently on cellular fitness (Fig 2C, D). For most drugs it was evident that the approximation of any single growth variable to fitness would overlook fundamental features of drug action; e.g. some chemicals resulted in similar reduction in growth rate but differed in their impact on the other two variables. However, although it is clear that different drugs tend to affect growth differently there is a correlation between drug impact on growth lag and growth rate (Fig 2C, linear correlation r^2 ^= 0.19). No such correlation was found between growth efficiency and growth rate (Fig 2D, linear correlation r^2^= 0.01). Interestingly, drugs which are structurally and chemically distinct but nevertheless target the same biological process displayed striking similarities in impact on cellular growth dynamics. One example is the well-established ergosterol biosynthesis inhibitors ketoconazole, clotrimazole and fenpropimorph which strongly reduced growth efficiency with only minor defects in growth rate and a slightly alleviating effect on growth lag. A similar fingerprint was found for the sphingolipid biosynthesis inhibitor aureobasidin A (ABA), suggesting that drugs targeting lipid metabolism primarily reduce growth efficiency. Strong effects on growth efficiency were also observed for the heavy metals Cd^2+ ^and Mn^2+^. Among the compounds that primarily affected growth lag were the two redox active agents DTT and diamide (Fig 2C, D). This suggests that drug induced perturbations of cellular redox status requires a time consuming reprogramming of the redox regulation system but causes little permanent damage. Finally, the two distinct DNA damaging agents present in the screen, the ribonucleotide reductase inhibitor hydroxyurea and the DNA methylating agent MMS, belonged to a small subset of compounds which specifically reduced growth rate while actually enhancing the capacity to quickly re-initiate growth. Taken together, the here reported results suggests that the impact of a drug on cellular growth dynamics is a consequence of its mode-of-action and that the three fundamental growth variables may be used as a high-resolution chemogenetic fingerprint of bioactive compounds.

### Cellular growth dynamics and gene-drug interactions

**Figure 3:**
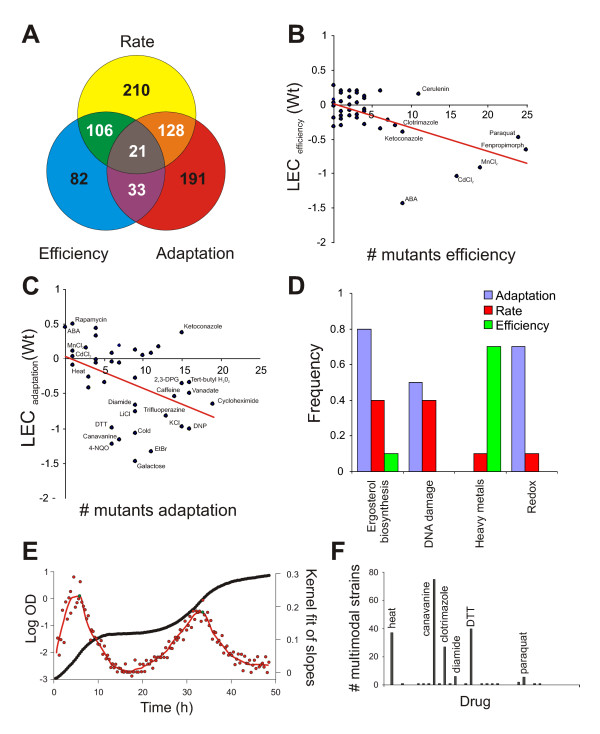
**Gene-drug interactions in different physiological windows**. A) Venn diagram depicting the number of significant growth defects (LPI < 0, p < 0.001) within a gene-drug mini-array. B-C) Comparing the relative growth reducing effect (LEC) of bioactive compounds on yeast WT populations to the number of knockouts displaying significantly reduced (LPI < 0, p < 0.001) tolerance to a specific compound. B) Considering growth efficiency (r^2 ^= 0.37). C) Considering growth lag (r^2 ^= 0.16). D) Frequency of clustering of bioactive compounds with similar mode-of-action (see results and discussion section). Repeated (n = 10) K-mean clusterings, in groups (k = 10) was performed and frequency of co-occurrence indicated. E) Drug induced multimodal growth in *tif3*Δ in cerulenin. Black circles = observed OD values, red circles = derivatives (slopes) of observed OD values, red line = smooth estimate, ${\tilde{f}}^{\prime }$, of the function that best fits the derivatives of the observed OD values, green circles = maxima in ${\tilde{f}}^{\prime }$. F) Number of knockouts for which a specific drug displays multimodality.

A central theme in chemical biology is to link chemicals' mode-of-action to the functionality of specific genes, i.e. to screen for gene-drug interactions. We analyzed the chemogenetic growth dynamics behavior of our 38 compounds in a mini-array of 96 gene knockouts. These mutants were selected as being generally stress sensitive and as involved in a wide diversity of functions like transcriptional regulation (e.g. *GTS1, MIG2*), detoxification (e.g. *PDR5*), DNA repair (e.g. *TOP1*,*RIS1*) and translation (e.g. *TIF2*, *TIF3*). Gene-by-drug interactions were precisely quantified as Logarithmic Phenotypic Indexes (LPI) [13], which provides a measure of non-multiplicative effects of combining a chemical and a genetic perturbation. The overlap between drug-gene interactions for the different growth variables was found to be limited (Fig 3A). Only for 21 (2%) of the 1080 recorded aggravating drug-gene interactions could we score an interaction in all three growth variables. The greatest overlap was observed between growth rate and growth efficiency; 53% of growth efficiency gene-drug interactions was also observed as growth rate interactions. The lowest overlap was observed between growth lag and growth efficiency; only 10% of growth lag defects were also detectable as growth efficiency defects. Thus, for many chemicals it was essential to follow the whole growth dynamic to score significant drug-gene interactions, and no single growth variable by itself provided a complete view of the chemogenetic interaction landscape. However, it should be noted that there was a statistically significant overlap between all variables, with the weakest overlap between efficiency and adaptation (Fisher's exact test, p < 9E-5). Second, we investigated whether the LEC values of a specific drug predict which growth variable most frequently captured gene-drug interactions for that drug. Statistically robust correlations (Fig 3B, C) was found considering either growth efficiency (linear regression, r^2 ^= 0.37) or growth lag (linear regression, r^2 ^= 0.16). Thus, drugs with a strong impact on growth efficiency in the wild type tended to show numerous growth efficiency gene-drug interactions whereas drugs that impacted strongly on growth lag frequently induced growth lag gene-drug interactions.

Bioactive compounds may be functionally grouped on the basis of similarities in growth rate chemogenetic profiles. However, such approaches can typically only cluster a minority of drugs known to be related. Our data on growth dynamics suggested that the insufficient power of clustering approaches partly can be explained by compounds being mainly affected on growth variables that are not resolved in the actual screen. To test this, repeated K-mean clusterings of the drugs in the gene-drug mini-array was performed, separately for each growth variable. The compounds known to be functionally linked and sharing mechanism of action, which we also could verify (Fig 2C, D), were used as a golden standard in this test: i) ergosterol biosynthesis inhibitors (clotrimazole, ketoconazole, fenpropimorph) ii) heavy metals (Cd^2+^, Mn^2+^) iii) redox-status distorters (DTT, diamide) iv) DNA damage inducers (MMS, hydroxyurea). Clustering the chemicals based on the drug-gene interactions from mutants phenotypes on the growth variable most affected in the wild type provided the most accurate functional grouping (Fig 3D): e.g. in the case of the azoles growth lag is clearly the growth variable that is most valuable in terms of clustering the three ergosterol biosynthetic inhibitors from gene-drug interaction data, and growth lag is also the most sensitive of the growth variables (Fig 2C, D). Accurate grouping of Cd^2+ ^and Mn^2+ ^was observed exclusively when clustering on growth efficiency, the growth variable most affected by these metal ions in the wild type. Hence, the growth variable primarily affected by a drug in the wild type also tended to be most revealing in terms of that chemical's functional implications from data on gene-drug interactions.

Interestingly, close scrutiny of the derived growth curves revealed that gene-drug interactions frequently were reflected not in aberrations of the three fundamental growth variables, but in the emergence of growth multimodality (Fig. 2E). To distinguish and objectively quantify the multimodality phenomenon, the growth curves in our gene-drug mini-array were subjected to mathematical modelling. A function was fitted to each growth curve by kernel smoothing; this function was derivatized and isotonic regression techniques were used to identify the presence of more than one function maxima (Fig 3E). Analyzing all individual gene-drug combinations we found 6% of the growth curves to be distinctly multimodal. Multimodality was never observed for unstressed mutants in basal medium, nor for approximately half of the 38 compounds. In contrast, the toxic arginine homolog canavanine induced multimodality in 80% of the knockouts whereas heat and clotrimazole displayed 40% multimodality (Fig 3F). The only additional compounds that induced multimodal growth in more than 5% of the knockouts were paraquat, diamide and DTT, drugs that all perturb cellular redox status. This implicates redox imbalance as one mechanism underlying multimodality. Our findings suggests that drug induced multimodality is a hallmark of a distinct set of drugs and that quantification of growth curve modality may increase the power of chemical fingerprinting.

### Cellular growth dynamics and drug-drug interactions

**Figure 4:**
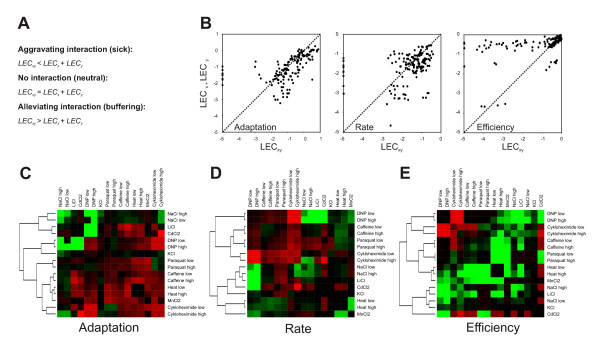
**Drug-drug interactions in different physiological windows**. Interactions within a mini-array of multi-replicated (n = 50 for single compounds, n = 20 for compound combinations) bioactive compounds. A) Multiplicative model of synthetic chemical interactions. B) Overview of all drug-drug interactions. Dashed line indicates null interaction, i.e. 1:1 correlation between observed (LEC_xy_) and expected (LEC_x _+ LEC_y_) effects. C-E) Heatmap of drug-drug interactions depicted as observed (LEC_xy_) – expected (LEC_x _+ LEC_y_) effects. Red = alleviation, green = aggravation.

In contrast to gene-gene and gene-drug interaction screening, which both have been extensively pursued, the potential of drug-drug interactions in deciphering mechanistic features of drug action have been poorly exploited. Only rather recently have the potential of large scale drug-drug screening received closer attention, particularly in the clinical context of multi-drug therapeutics [10,15]. To investigate drug-drug interactions in the light of the differential drug impact on growth dynamics a subset of the here used bioactive compounds was screened using a combinatorial array design. The growth perturbing effect (LEC) of each individual compound and each combination of compounds was quantified. We applied a standard multiplicative model to predict no synthetic drug interactions. In this model, no interaction between two compounds assumes that the growth defects arising from the combined application of two compounds, LEC_xy_, equals the calculated sum of the growth defects of each individual compound, LEC_x _+ LEC_y _(Fig 4A). We observed frequent aggravating and alleviating interactions for all three growth variables (Fig 4B). In total, 32% of the drug-drug interactions, alleviating or aggravating, would be overlooked if growth rate were used as sole phenotypic measure. Moreover, whereas alleviation were substantially more frequent considering growth lag (2.9 fold more common) and growth rate (1.8 more common), aggravating drug-drug interactions dominated for growth efficiency (2.6 fold more common). The high frequency of growth efficiency drug-drug synergism is interesting considering that aggravating interactions are most informative for interpretations of drug mode-of-action. As one example, the redoxcycler paraquat displayed an aggravating interaction with the heavy metals Cd^2+ ^and Mn^2+ ^exclusively on the level of growth efficiency (Fig 4C). Heavy metals are indeed thought to exert chemotoxicity primarily by inducing oxidative stress [16]. Interestingly, many of the observed growth efficiency drug-drug interactions could not be predicted on the basis of the effect of the individual compounds on cellular growth dynamics in the wild type (Fig 2). For example, the chemically related Na^+ ^and Li^+ ^only weakly reduced growth efficiency on their own, but featured a strongly aggravating growth efficiency interaction when combined. This is in line with the assumption that Li^+ ^mimics Na^+ ^with regards to the effect on biological systems [17]. We also noted that addition of the protein synthesis inhibitor cykloheximide alleviated the effects of many drugs, e.g. DNP (Fig 4C), indicating that drug toxicity, in many cases, is dependent on an unperturbed protein production.

In previous chemogenetic screens, only partial consistency between specific chemical synergies was revealed [15]. The here reported phenotypic differences between physiological windows suggest that some of the disagreement may be because diverse phenotypic outputs are grouped together. For example, colony based screening assays analyze a composite of all the three growth variables in addition to a colony competition factor due to that neighbours compete for nutrients in the same portion of solidified medium. Given the here reported results, it is not surprising that drug-drug interactions scored using a colony size assessing screening system should diverge substantially from interactions derived using screening systems exclusively quantifying growth rate. In conclusion, the differences in drug-drug interaction patterns observed between different growth variables underscores the importance and value of resolving all three growth variables when studying the chemotoxic effects of bioactive compounds using cell arrays.

## Conclusion

Taken together, the here reported results show that the power of chemogenetic approaches may be increased by resolving growth into its individual components. Increasing physiological depth and thereby phenotypic space is of pharmacological importance as elucidation of drug function relies heavily on the ability to elicit a rich range of phenotypes, especially in terms of quantifying off-target effects. Thus, by facilitating a physiologically more complete analysis of gene-drug and drug-drug interactions the here reported results high-light the potential of high resolution micro-cultivation and the analysis of growth dynamics for pharmacological use in characterizing orphan bioactive compounds.

## Methods

### Strains

Knockout strains in the BY4741 background [18] were provided by the EUROSCARF stock centre . WT genotype: *MATa;his3Δ1;leu2Δ0;met15Δ0;ura3*, knockout genotype:*MATa;his3Δ1;leu2Δ0;met15Δ0;ura3, ORF*::*kanMX4*.

### Cultivation and drug concentrations

Pre-cultivation (two serial rounds) and cultivation in a Synthetically Defined (SD) medium, with and w/o drugs, were performed as earlier described [13]. For initial testing of drug dose-response correlations, a ladder of concentrations selected as to encompass concentrations used in published studies, were chosen. On the basis of drug dose-responses, concentrations for the gene-drug mini-array and the drug-drug mini-array were set as to enable reliable quantification of all three fitness variables. Concentrations for the gene-drug mini-array were: o-Phenanthroline 0.2 μM, 2,3 Diphosphoglycerate 13 mM, 2,4 Dinitrophenol 0.2 mg/ml, 4-NQO (Nitroquinolone) 0.8 μg/ml, 6-Azauracil 200 μg/ml, AT-3 (1,2,4-Aminotriazole) 315 mM, AureobasidinA 2.5 μg/ml, Caffeine 0.65 mg/ml, Canavanine 0.5 μg/ml, CdCl_2 _47.5 μM, Cerulenin 0.22 μg/ml, Clotrimazole 1.5 μM, Coldstress 19°C, Cykloheximide 0.035 μg/ml, Diamide 1.4 mM, DMSO (Dimethylsulfonyloxide) 1,5%, DTT 1.6 mM, Ethidium bromide 45 μg/ml, Fenpropimorph 0.05 mg/ml, Galactose 2% (as sole carbon source), Heatstress 40°C, Hydroxyurea 8 mg/ml, Hygromycin B 100 μg/ml, KCl 1.45 M, Ketoconazole 20 μM, LiCl 100 mM, Methyl Methane Sulphonate (MMS) 0.0015%, MnCl_2 _10 mM, Myriocin 2 μg/ml, NaCl 0.85 M, Neomycin 2 mM, Paraquat 200 μg/ml, Rapamycin 0.3 μg/ml, tert butyl-OOH 0.35 mM, Thiabendazole 0.06 μg/ml, Trifluoperazine 25 μM, Tunicamycin 1 μg/ml, Sodium-ortho vanadate 1.45 mM).

### Growth analysis

To quantify the effect of bioactive compounds on different WT growth variables eight wild-types (WT) were cultivated in ten drug concentrations. WT growth in 30C (except where otherwise stated) was measured using a Bioscreen Analyzer C (Growth Curve Oy, Finland) as earlier described [13]. Measurements of Optical Density (OD) was taken every 20 min during a 48 h period (72 h for the drug-drug mini-array) resulting in growth curves. For each growth curve the growth variables growth rate, growth efficiency and growth lag were calculated was as earlier described [13]. Growth curves which, due to no or very poor growth, could not be reliably dissected by the automated procedure were manually inspected and best estimate growth variable measures were extracted subjectively if relevant. For each growth variable and each compound a Logarithmic Environmental Coefficient, LEC, was formed as:

$${\text{LEC}}_{j}=\left[\left[\frac{1}{8}\sum_{k=1}^{8}\log\left(wt_{knormal}^{r}\right)\right]-\left[\frac{1}{8}\sum_{k=1}^{8}\log\left(wt_{kj}^{r}\right)\right]\right]$$

where *wt*_*kj *_is the growth variable of the *k*:th growth curve of the wildtype in drug *j*, *wt*_*knormal *_is the the growth variable of the *k*:th growth curve of the wildtype in normal (no stress) condition and *r *indicates the run. For growth efficiency the *wt*_*kj *_and the *wt*_*knormal*_expressions were reversed; hence, for all growth variables a negative LEC indicates a growth reducing effect of the drug.

To quantify the drug tolerance of gene knockouts (n = 2) as compared to WT (n = 8) in the gene-drug growth curves and growth variables were derived as above. For each growth variable in each drug a Logarithmic Strain Coefficient, LSC, was formed as earlier described [13]. Briefly, the LSC measure may be thought of as the log ratio LN (WT/knockout). Furthermore, to distinguish drug tolerance from growth in no stress conditions a Logarithmic Phenotypic Index, LPI, was formed for each knockout in each drug, also as earlier described [13]. The LPI measure may essentially be thought of as LSC_drug _– LSC_no stress_, hence a negative LPI indicates a reduction in the tolerance of a specific gene knockout to a specific drug. We performed tests of the null hypothesis that LPI equals 0 separately for each knockout population and chemical stressor and separately for each fitness variable. Statistical significance was calculated using a threshold of three mean standard deviations. In order not to reject the null hypothesis only because of single extreme value, a two-tailed, two sample Students T-test (α < 0.05, df = 2) was also applied. These combined measures gave a significance level of α < 0.001 (Gaussian distribution and equal variance assumption). Genes included in the screen were: *FLO1, PIM1, TAT1, YBR074w, PHO3, YBR099c, APE3, APM3, YCL010C, YCL047C, CVT17, YCR073W, SRB8, YCR101C, YCR106W, YCR195C, YDL109c, YDL124w, DLD1, YDL175c, UGA4, GDH2, RRI1, SHS1, YDR026c, YDR101c, YDR132c, SWM1, GLO2, SUM1, YDR384c, HAT2, PRB1, CAN1, VTC1, DOT6, FTR1, YGL010w, ATE1, YGL131c, YGL144c, AMS1, APG1, GTS1, YGL196w, MIG2, KIP3, EDC1, YGL242c, HFM1, HXK2, BNS1, YHL002w, YHL029C, SOD2, PCL7, SPO22, UBP7, MPH1, HYR1, YJL131c, TIF2, YJR044c, SOD1, MNN4, EAP1, YKR090w, YKR104w, YBT1, AYT1, DAN2, APC9, YLR108c, PDC5, TFS1, YLR422w, YLR426w, YAP1, MSC1, STV1, SIP18, SIW14, YNL056w, TPM1, YNL099c, TOP1, GSH2, HST1, NDJ1, MDH2, WHI2, PDR5, RIS1, EAF3, YPR139c *and *TIF3*.

### Mathematical modeling of multimodal growth

For the gene-drug mini-array, multimodal growth was analyzed for each growth curve separately. The measurement of the OD-value y_i _= y(t_i_) at time point t_i _can be described as *y*(*t*_*i*_) = *f*(*t*_*i*_) + *ε*_*i *_y(t_i_) (1) where the function *f *is the theoretical growth curve. The term *ε*_*i *_describes the deviation from the population mean due to biological variation. The theoretical growth curve *f *is assumed to consist of one or two sigmoidal parts; a sigmoidal part being an interval on which the function f is first convex, i.e. *f" *> 0, and then concave, i.e. *f" *< 0. Note that *f *being sigmoidal means that the slope of the growth curve *f' *is first increasing, so that (*f'*)*' *= *f" *> 0, and then decreasing, so that (*f'*)*' *= *f" *< 0. Thus, that *f *consist of a single sigmoidal part is equivalent to the slope of the growth curve *f' *being unimodal. Similarly, that *f *consists of two sigmoidal parts is equivalent to *f' *being bimodal. Thus we can make the equivalent assumption on the theoretical growth curve *f *that its derivative *f' *is either unimodal or bimodal. Using the equivalence between sigmoidality of f and unimodality/bimodality of *f' *we obtain an alternative biological model.

Let ${y^{\prime }}_{1}=\frac{y_{i}-y_{i-1}}{t_{i}-t_{i-1}}$ be the observed slopes in the OD values. Then from (1) follows the biological model: *y' *(*t*_*i*_) = *f' *(*t*_*i*_) + *ε*_*i *_(2). Here *f' *is the derivative of the mean growth curve. Given measurements (*y*_*i*_, *t*_*i *_y_i_, t_i_) of OD values assumed to follow the biological model (1), we want to estimate the (unknown) mean growth curve *f *under the assumption that it consists of one or two sigmoidal parts. Let *F *denote the set of all functions that consist of one or two sigmoidal parts. Then an estimate $\hat{f}$ of *f *can be obtained by minimizing the least squares error between the observed OD values *y*_*i *_and the mean OD values *f*(*t*_*i*_) over the set of all functions in *F*. There is to our knowledge no analytic solution to this problem [19]. We therefore make a slight modification of the estimation approach. Let *F' *denote the set of functions that are unimodal or bimodal, i.e. containing all derivatives *f' *of the mean growth curve *f*. Then an estimate ${\hat{f}}^{\prime }$ of *f' *can be obtained by minimizing the least squares error between the observed slopes of OD values *y' *and the mean slopes of OD values *f' *(*t*_*i*_) over the set of all functions in *F' *. However, there is no analytic solution even to this problem. We therefore simplify the approach further. First, we smooth the data ${y^{\prime }}_{1}$ using a kernel smoother [20,21] to obtain a smooth estimate ${\tilde{f}}^{\prime }$ of *f' *. We use ${\tilde{f}}^{\prime }$ to obtain an estimate of the first mode as the position *m*_1 _where ${\tilde{f}}^{\prime }$ is maximal. Second, we fit a unimodal function ${{\hat{f}}^{\prime }}_{1}$ with mode at *m*_1 _to the data (*t*_*i *_${\tilde{f}}^{\prime }$ (*t*_*i*_)) as the function that minimizes the sum of squares between ${\tilde{f}}^{\prime }$ and ${\hat{f}}^{\prime }$[22,23]. We can decide on whether the curve is bimodal or not by looking at the maximal difference ${\tilde{f}}^{\prime }$ - ${\hat{f}}^{\prime }$; if this maximal difference is positive we classify the curve as bimodal, if it is zero we classify it as unimodal. From the growth curve estimating algorithm we can get a multimodality parameter D such that D = 1 when the curve is classified as bimodal and D = 0 when the curve is classified as unimodal. In order to minimize the risk for obtaining false positives, we use bootstrap techniques [24]. Thus, for each experiment we draw N random samples from the residuals in the model (1) which we add to the estimated theoretical curves $\hat{f}$ to obtain N bootstrap growth curves; for each of these we estimate the parameter D to obtain bootstrap estimates $D_{1}^{*}...D_{N}^{*}$. We use the proportion of $D_{i}^{*}$ = 1 to obtain an estimate p* of P(D = 1). If p* is close to one (p* ≥ 0.8) we classify the growth curve as bimodal. Conservatively, an experiment (gene-environment combination) is classified as bimodal, when, and only when, both replicate growth curves display bimodality.

## Authors' contributions

JW designed and performed most of the experimental work and the data analysis. DA performed the analysis of multimodal growth. BL performed experimental work on dose-reponse correlations. AB designed, coordinated and supervised the project. JW drafted the manuscript with substantial contributions from AB. All authors participated in the revision of, and have given approval to, the final version of the manuscript.
